# Assessment of the Association Between Lung POCUS Findings During Preoperative Assessment and Cardiopulmonary Outcomes in Patients Undergoing Major Abdominal Surgery: A Pilot Study Protocol

**DOI:** 10.24908/pocusj.v10i02.18912

**Published:** 2025-11-17

**Authors:** Leonidas Palaiodimos, Sriram Sunil Kumar, Perminder Gulani, Maisha Maliha, Adam Mylonakis, Lori Lemberg, Mindaugas Pranevicius, Robert T. Faillace, Ilias I. Siembos, Benjamin Galen, Dimitrios Schizas

**Affiliations:** 1 Department of Medicine, Jacobi Medical Center, Bronx, New York, USA; 2 Albert Einstein College of Medicine, Bronx, New York, USA; 3 First Department of Surgery, National and Kapodistrian University of Athens, Laikon General Hospital, Athens, Greece; 4 Department of Anesthesia, Jacobi Medical Center, Bronx, New York, USA; 5 First Department of Critical Care Medicine and Pulmonary Services, National and Kapodistrian University of Athens, Evangelismos Hospital, Athens, Greece; 6 Department of Medicine, Montefiore Medical Center, Bronx, New York, USA

**Keywords:** Point of care ultrasound (POCUS), Noncardiac surgery, Lung POCUS, Peri-operative

## Abstract

Abdominal surgeries make up a significant portion of all surgical procedures performed worldwide. Despite advances in surgical techniques, there is significant morbidity and mortality associated with abdominal surgeries. Cardiopulmonary complications in the postoperative period play an important part in the elevated risk associated with these surgeries. Preoperative medical assessments have therefore become the standard of care to evaluate the risk of surgery, optimize a patient's medical conditions, and mitigate the perioperative risk. While there has been increasing utilization of lung point of care ultrasound (POCUS) in the immediate preoperative setting, the use of lung POCUS at the preoperative medical assessment clinic visit has not been studied. While using risk stratification tools is common in current practice, the role of adjunctive office-based techniques like lung POCUS have not been studied in this setting. We conducted an observational prospective pilot study to evaluate the association of lung POCUS findings in the preoperative visit on the risk of adverse cardiopulmonary outcomes in the 30-day postoperative period after major abdominal surgery. A standardized scoring system called integrated lung ultrasound score (iLUS) is used for objective assessment. Our study attempted to determine whether the addition of lung POCUS can be used to better stratify the risk for postoperative complications.

## Introduction

Each year, more than 300 million patients receive a major surgery worldwide; around 50 million in the Unites States and 20 million in Europe [1–3]. Abdominal surgeries consist of about 15% of all surgeries [4]. Despite the tremendous advances and overall outcome improvement in surgery, the perioperative morbidity and mortality remain significant. A study based on the American College of Surgeons National Surgery Quality Improvement Program database estimated 30-day postoperative mortality rates by surgical urgency: 0.4% for elective procedures, 2.3% for urgent surgeries, and 3.7% for emergent cases. In contrast, 30-day morbidity rates were remarkably higher, reaching 6.7%, 12.3%, and 13.8%, respectively [5].

Cardiovascular complications, mainly myocardial ischemia, left ventricular dysfunction, and arrhythmia—occur in one in seven patients older than 45 years within 30 days from an inpatient non-cardiac surgery [6]. Similarly, pulmonary complications are common—particularly after major abdominal surgery—with an estimated incidence of 9 to 40%. Atelectasis, pneumonia, and pulmonary aspiration are among the most common postoperative complications and can impact survival after surgery. Preoperative medical assessment prior to every major surgery has become the standard of care with objectives to assess the perioperative risk for morbidity and mortality, optimize the patient's medical condition, and mitigate the risk for cardiopulmonary complications and others [9]. A number of validated risk stratification tools, such as the Revised Cardiac Risk Index (RCRI), American University of Beirut (AUB)-HAS2 Cardiovascular Risk Index, the American Society of Anesthesiologist's Physical Status (ASA-PS), and the Gupta perioperative risk for myocardial infarction and cardiac arrest have been created and used to standardize and optimize the perioperative medical assessment process [10–12]. The utilization of point of care ultrasound (POCUS)—the application of portable ultrasound by clinicians at the bedside—has grown rapidly in the inpatient and outpatient settings and is a valuable tool that enhances the standard clinical assessment [13]. In particular, lung POCUS can reveal findings, such as mild pulmonary edema, atelectasis, fibrosis, or small pleural effusions, which can be subclinical and otherwise not detected by history and physical examination alone [14]. A structured approach to performing and evaluating lung POCUS has been highly useful. This was originally known as the lung ultrasound score, and was later refined by Dell'Aquila et al. as the integrated lung ultrasound score (iLUS) [15,16].

Studies conducted in the immediate preoperative period have shown that lung POCUS performed during anesthesiology evaluation can aid in determining fluid therapy and predicting major adverse cardiac events [17,18]. However, the specific role of POCUS beyond this immediate preoperative assessment of patients has not been studied adequately, especially in the clinic setting at the time of preoperative medical assessment [19].

## Aims and Hypothesis

We aim to study the value of adjunctive iLUS at the time of preoperative medical assessment by internal medicine physicians on postoperative cardiopulmonary outcomes. Our primary hypothesis is that early lung POCUS assessment in moderate- to high-risk adult patients over 50-years-old undergoing abdominal surgery can reduce postoperative complications, thereby decreasing intensive care unit admissions, mortality, hospital length of stay, and readmissions.

## Methods

### Study design and setting

This observational prospective pilot study will be conducted in New York City Health + Hospital (NYC H+H)/Jacobi, New York, United States of America affiliated to Albert Einstein College of Medicine and Laikon General Hospital, Athens, Greece affiliated to National and Kapodistrian University of Athens. The study has been reviewed and approved from Biomedical Research Alliance of New York (BRANY) and the respective NYC H+H committee, as well as the ethics committee of Laikon General Hospital. Verbal informed consent will be obtained from all participants. Written consent was waived as POCUS is the standard of care and an integral part of physical examination in modern clinical medicine.

### Population, Inclusion and Exclusion Criteria

We plan to include 120 men and women (at least 20 of them from the Laikon site) with the inclusion criteria consisting of patients ≥50 years of age, who undergo preoperative medical assessment for a major abdominal surgery under general anesthesia, have RCRI ≥2 (moderate or higher risk), and ASA-PS: II, III, IV (Table 1 and 2).

**Table 1.** Revised Cardiac Risk Index (RCRI) for preoperative risk

RCRI clinical variable	Points
Elevated-risk surgery (intraperitoneal, intrathoracic, suprainguinal vascular)	1
History of ischemic heart disease	1
History of congenital heart failure	1
History of cerebrovascular disease	1
Preoperative treatment with insulin	1
Preoperative creatinine >2 mg/dL	1

**Table 2.** American Society of Anesthesiologist's Physical Status (ASA-PS) - ASA classification

ASA Classification	Definition
ASA I	Normal healthy patient
ASA II	Patient with mild systemic disease
ASA III	Patient with severe systemic disease
ASA IV	Patient with severe systemic disease that is constant threat to life
ASA V	Moribund patient not expected to survive without operation
ASA VI	Declared brain dead patient whose organs are being removed for donor purposes

The exclusion criteria will be age <50 years, surgery other than major abdominal, anesthesia other than general, RCRI <2 points, and ASA-PS other than 2, 3, or 4.

### Intervention

Included patients will undergo lung POCUS examination as part of their preoperative medical assessment prior to surgery. This is typically performed by an internal medicine specialist, and is usually done in a week to a few days in an outpatient clinic (in case of elective surgeries), and inpatient 0-1 days prior (in case of urgent surgeries). The studies will be performed by a sole operator (author LP), who was trained in POCUS during his internal medicine residency program, has attended POCUS courses via the American College of Physicians, and has been using POCUS on a daily basis in his clinical practice for more than five years. A single operator was chosen to eliminate the impact of inter-operator variability. POCUS images will be saved. To validate the findings, the operator's interpretation will be confirmed by an independent reviewer, who is a POCUS-trained critical care attending physician at NYC H+H/Jacobi (author PG). Clinicians will be blinded to lung POCUS findings, which is in-line with the high-quality POCUS literature [18]. Exceptions will include POCUS findings that are considered by the operator as critical, such as possible lung collapse, severe pulmonary edema, or large pericardial effusion, which will be reported to the clinicians.

**Figure 1.**
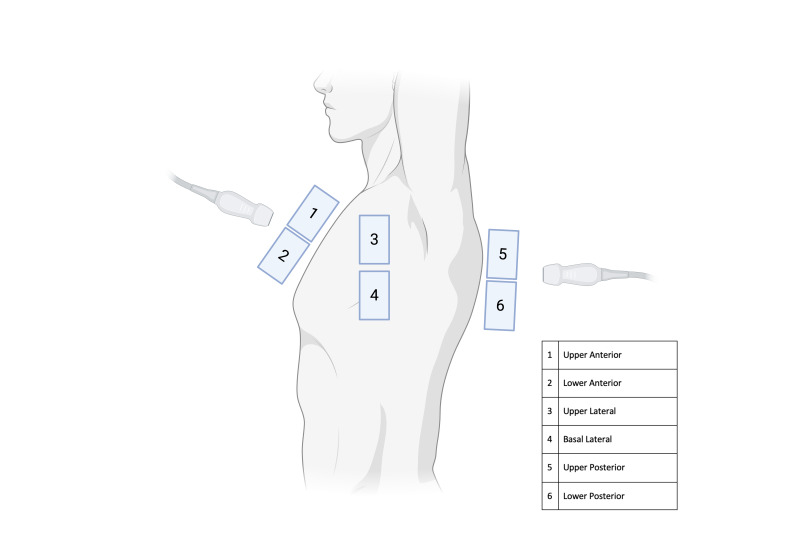
The six chest areas per side considered for complete 12-zone lung POCUS examination. Areas 1 and 2 denote the upper anterior and lower anterior chest areas, respectively. Areas 3 and 4 denote the upper lateral and basal lateral chest areas, respectively. Areas 5 and 6 denote the upper posterior and lower posterior chest areas, respectively [31].

A commercial handheld device will be used on its lung preset. The 12-zone lung POCUS examination will be performed (Figure 1). The iLUS will be calculated for every patient. The external lung fields will be examined dividing the surface of the thorax into 12 zones: 6 on the right (anterior: upper R1 and lower R2; lateral: upper R3 and lower R4; posterior: upper R5 and lower R6) and 6 on the left side (anterior: upper L1 and lower L2; lateral: upper L3 and lower L4; posterior: upper L5 and lower L6) (Figure 1). iLUS assigns 0 points to A-lines or?<2 separate B-lines plus regular sliding; 1 point with B-lines?≥3 or spaced focal points plus regular sliding; 2 points with coalescing B-lines, and 3 points to pulmonary consolidations with a score ranging from 0 (normal lungs) to 36 (worst case scenario). iLUS evaluation then integrates four additional parameters: i) presence of pleural effusion (value 0 if absent, value 1 if present), ii) presence of pericardial effusions (value 0 absent, value 1 present), iii) measurement of the inferior vena cava (IVC) respiratory variation (<33%) (value 0 if absent, value 1 if present), iv) diaphragm excursion. This last parameter will be measured during normal breathing via a right subcostal scan. An excursion?>2?±?0.5 cm will be considered normal (value 0 if absent), while a value below will be abnormal (value 1 if present). Therefore, the total value of the iLUS will be 40 (Table 3) [15].

**Table 3.** Integrated Lung Ultrasound Scoring (iLUS) system

Finding	Points
A0. A-line pattern or <2 B-lines with normal lung sliding	0
A1. ≥3 well-spaced B-lines	1
A2. Coalescing B-lines	2
A3. Pulmonary consolidation	3
B. Present pleural effusion	1
C. Present pericardial effusion	1
D. IVC variation <33%	1
E. Diaphragm excursion <2?±?0.5 cm	1

### Primary and Secondary Endpoints

The primary endpoint will be the composite of failure of extubation after surgery completion in the operation room with the need for re-intubation, unplanned use of continuous positive airway pressure (CPAP), bilevel positive airway pressure (BiPAP), or high-flow nasal cannula (HFNC) after postoperative extubation, admission to the intensive care unit for a primary cardiopulmonary etiology in the index admission, death from a primary cardiopulmonary etiology within 30 days from operation, or readmission for a primary cardiopulmonary etiology within 30 days from operation (whichever happens first). The secondary endpoints will be the individual components of the primary endpoint as above, the length of stay for patients discharged alive, the need for supplemental oxygen on post-operation day 1 in the surgery floor and the need for additional diagnostic tests or other interventions prior to the planned surgery as a response to critical POCUS findings. Length of stay is defined as the number of days from the day of the operation to the day when the patient is declared medically appropriate for discharge by the primary team.

### Participant follow-up and study duration

**Figure 2.**
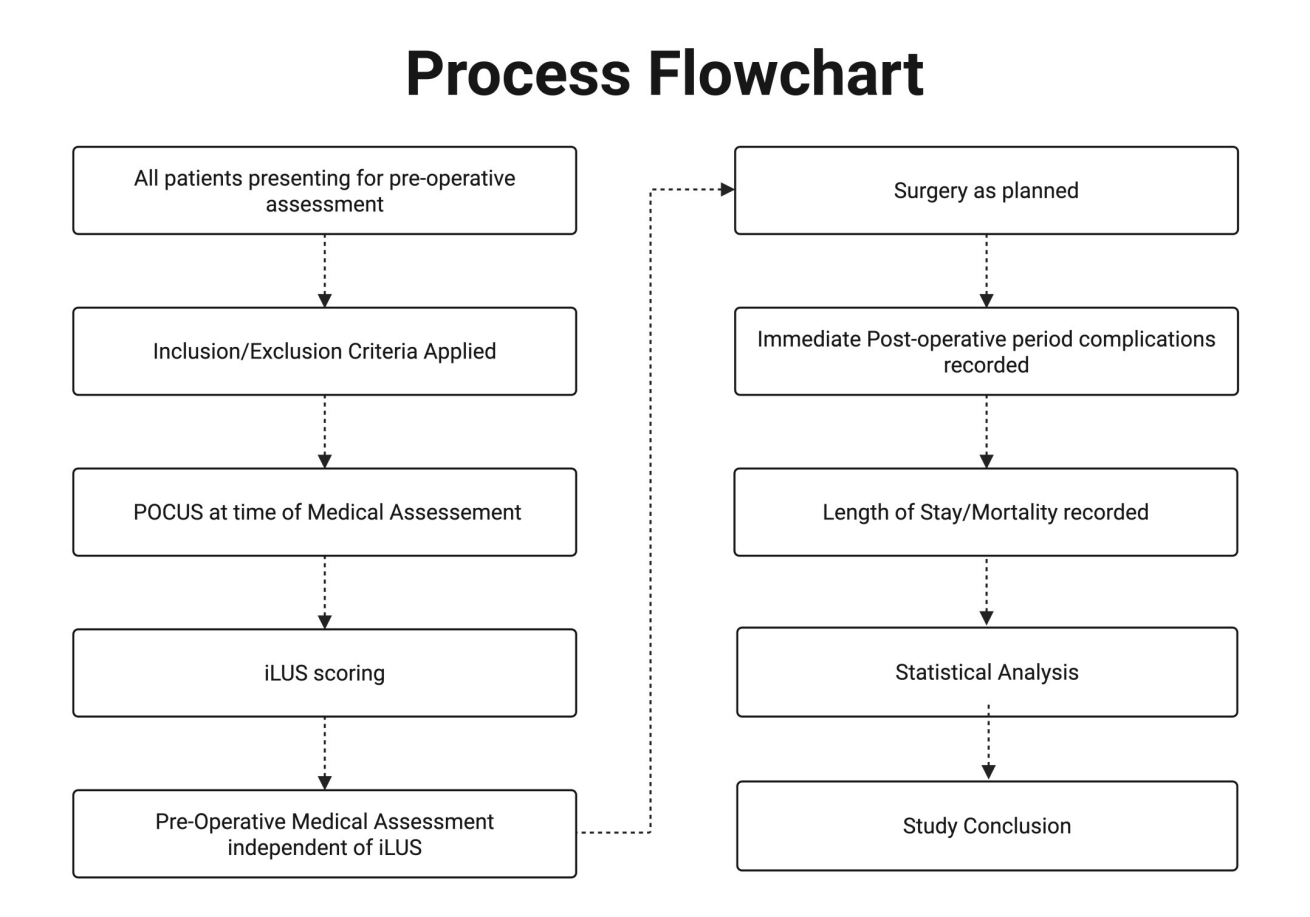
Process flowchart of the study

Participants will be followed for 30 days from the day of surgery. Postoperative complications after laparotomy are most common within a median of 9 days after surgery. Therefore, a follow up period of 30 days ensures that all acute cardiopulmonary complications are included [20]. The study is expected to be completed within two years after its initiation. The patient flow from intake into the study until completion of the study is demonstrated in Figure 2.

### Safety, Adverse Events Definition, and Reporting

Ultrasound imaging has been used for over 30 years and has an excellent safety record. It is based on non-ionizing radiation, so it does not have the same risks as X-rays or other types of imaging systems that use ionizing radiation [21]. POCUS has become standard practice in various aspects of daily clinical practice [22]. No adverse events are expected from POCUS, and this study poses no more than minimal risk for the included patients. POCUS findings that are considered by the operator as critical, such as possible lung collapse, severe pulmonary edema, or large pericardial effusion, will be reported to the primary team.

### Methodology of Analysis

The following 3 classifications of patients will be performed based on the iLUS: i) iLUS 0 vs. >0, ii) iLUS < mean iLUS vs. ≥ mean iLUS, iii) iLUS < median iLUS vs. ≥ median iLUS.

Continuous variables will be presented as mean with standard deviation and median with interquartile range. Categorical variables will be presented in absolute and relative frequencies. The appropriate parametric and non-parametric tests will be utilized for generating comparisons.

A sensitivity analysis will be performed for patients enrolled in the Jacobi site and patients enrolled in the Laikon site. Sensitivity analyses will be performed for various patient groups based on RCRI, ASA-PS, and AUB-HAS-2 scores, as well as whether the surgery was emergent or elective as deemed by the primary surgical team [23].

Multivariate analysis will be performed including iLUS classification and the following variables: age, sex, body mass index (BMI), RCRI, ASA-PS, and AUB-HAS-2 scores, time from POCUS scan to surgery, and emergent vs. elective surgery.

In addition, to preserve the integrity of our analysis while still reporting critical lung POCUS results, we will implement the following analysis: All patients enrolled in the study will be included in the primary analysis based on the intention-to-treat (ITT) principle to ensure that our results will reflect real-world practices. Patients with critical POCUS findings prompting interventions, postponement, or cancellation of surgery will be retained in the ITT analysis to maintain randomization integrity. Additionally, a pre-specified subgroup analysis will focus exclusively on these patients to evaluate the specific impact of the critical POCUS results on outcomes such as completion of surgery, postoperative complications, intensive care unit admission, mortality, length of stay, and readmissions. To further assess the robustness of our findings, a sensitivity analysis will be conducted, excluding patients with critical findings to evaluate the effect of blinding on the study outcomes.

An interim analysis will be performed after the follow-up completion of 60 participants (with at least 10 participants at the Laikon site). Should the interim analysis reveal findings that decisively confirm or reject the study hypothesis, no more participants will be enrolled, and the study will be ended after the follow-up completion of the last enrolled participant.

The statistical significance threshold will be set at p ≤0.05. Analysis will be conducted with STATA software (version 14.1, STATA corporation, College Station, TX, USA).

## Discussion

There is an emerging role of POCUS in the perioperative setting with its use by anesthesiologists in operative rooms for regional anesthesia and line insertion [24]. The use of cardiac POCUS in the preoperative evaluation of patients with femur neck fracture repair was found to show a significant decrease in both 30-day and 1-year mortality [25]. A similar demonstration of the utility and feasibility of preoperative-focused ultrasound was demonstrated by Cowie, however, most of the assessments occurred immediately prior to surgery [26]. This increasing use of perioperative POCUS is well-established among anesthesiologists in other contexts as well, such as in the setting of difficult laryngoscopies and for assessment of intra-abdominal fluid extravasation after hip surgery [27]. However, the utilization of lung POCUS in the preoperative medical setting is limited, as well as the respective research. Incorporating early lung POCUS into preoperative medical assessments will provide valuable additional context for optimizing both pre- and perioperative care. This approach may guide medication adjustments, potential postponement, or cancellation of surgery based on findings, enabling internal medicine physicians to offer more informed recommendations to surgical and anesthesia colleagues regarding medical risks.

Current guidelines only recommend adjunctive workup with electrocardiogram and echocardiograms in patients who have specific risk factors [28]. The scope of POCUS as an adjunctive assessment tool for cardiac or lung dysfunction has not been described in preoperative guidelines [29]. While there are studies evaluating the impact of lung POCUS examination by anesthesiologists in the perioperative period, the impact of lung POCUS by internal medicine specialists at the time of preoperative medical assessments has not been well described [30]. Therefore, this pilot study was designed to evaluate the possible role of lung POCUS in preoperative medical evaluation and the possible association of its findings with postoperative cardiopulmonary outcomes.

## Strengths

The study will explore an understudied topic on POCUS literature where research data is needed. This will be a real-world pragmatic study that will be conducted in two public hospitals in the Bronx, New York and in Athens, Greece, including patient populations that are typically underrepresented in research. All included patients will undergo lung POCUS by the same POCUS-trained clinician, and all images will be reviewed and validated by another POCUS-trained physician ensuring quality of acquired data. The lung POCUS protocol that will be used is one of the most comprehensive ones described in literature [15].

## Limitations

While this is a pilot study, subtle but clinically relevant associations between the lung POCUS findings and outcomes may be missed. Additionally, even though we have included two sites, there may be limited generalization considering that most patients will be from a single center. Moreover, our study will not evaluate the role of lung POCUS on low-risk patients or in patients undergoing non-abdominal surgeries.

## Conclusion

Our study will attempt to shed light on the role of lung POCUS in the preoperative clinic and its impact on patient outcomes, as well as to pave the way for more research on preoperative POCUS. While this will be a small pilot study, the results from the study can be used to inform the design of larger randomized controlled trials and subsequently affect guidelines on preoperative medicine.
